# Supplementary material for: Complex microparticle architectures from stimuli-responsive intrinsically disordered proteins
Source: Nat Commun. 2020 Mar 12;11:1342. doi: 10.1038/s41467-020-15128-9 (PMC7067844; doi:10.1038/s41467-020-15128-9)
Supplement: Supplementary file 1 — Supplementary Information [file 41467_2020_15128_MOESM1_ESM.pdf]

Supplementary Information

Complex Microparticle Architectures from Stimuli-Responsive Intrinsically Disordered Proteins

Roberts et al.

Supplementary Figures:

Supplementary Figure 1: Tuning ELP and POP T_{cp}'s 3
Supplementary Figure 2: Purity of protein polymers 4
Supplementary Figure 3: Control of ELP globule size in bulk mixtures 5
Supplementary Figure 4: Bulk images of hollow shell network 6
Supplementary Figure 5: Tunable sustained release of ELP from stable POP scaffolds 7
Supplementary Figure 6: Additional widefield images for microdroplet architectures 8
Supplementary Figure 7: POP particle shrinking and swelling 9
Supplementary Figure 8: 12.5% POP Particles 10
Supplementary Figure 9: Mixtures of ELP and a more hydrophobic POP 11
Supplementary Figure 10: Unnatural amino acids (UAA) for UV crosslinking 12
Supplementary Figure 11: UV exposure for xPOP microparticles 13
Supplementary Figure 12: xPOP mixture with a hydrophilic ELP 14
Supplementary Figure 14: ELP coacervation within POP shells 16
Supplementary Figure 15: Confocal reconstructions of hollow protein architectures 17
Supplementary Figure 16: Young's Modulus (E) of planar gels and POP microparticles 18
Supplementary Table 1: Polymer Sequences 19

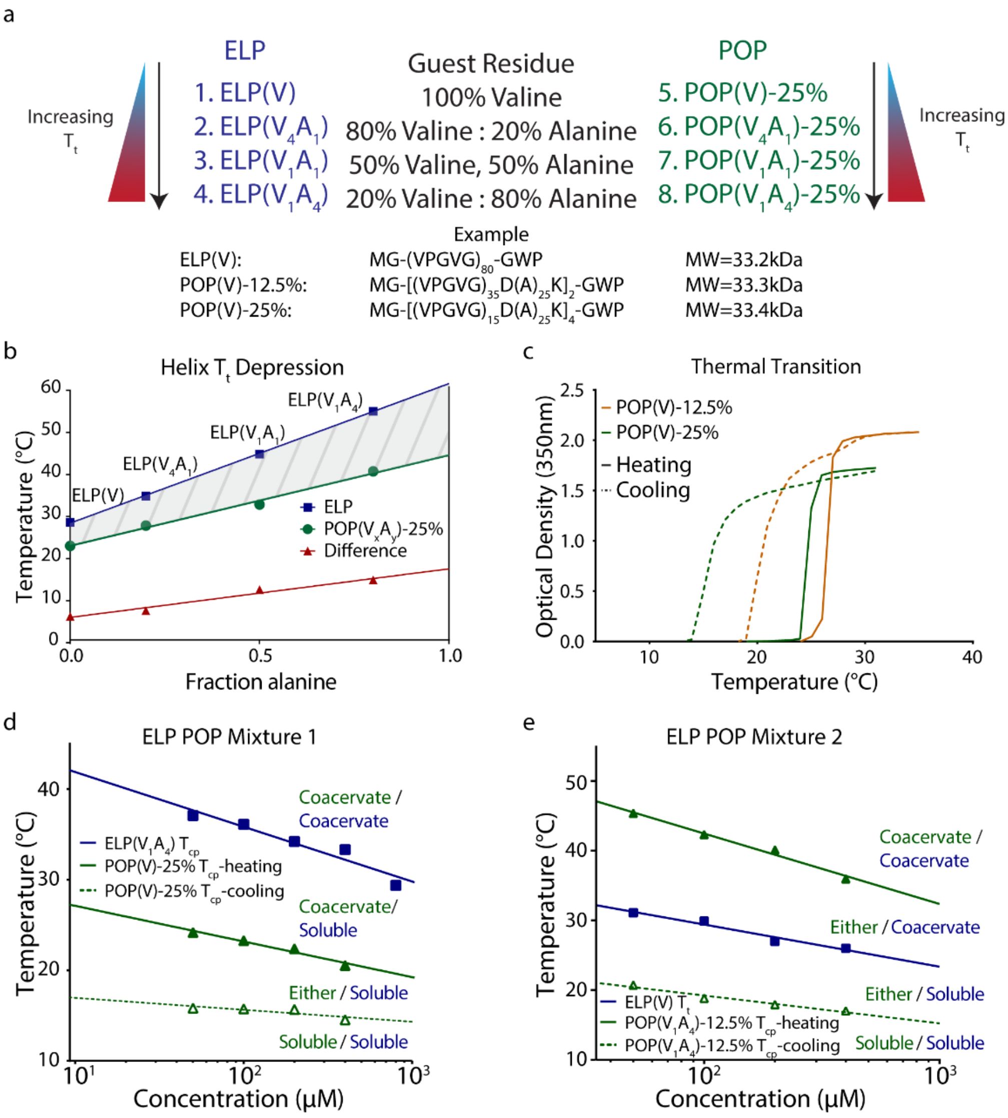

Supplementary Figure 1: Tuning ELP and POP T_{cp}'s. (a) Polymer sequences and notation used within the manuscript. ELPs and POP with different ratios of valine (V) and alanine (A) were used to tune polymer transition temperatures (T_{cp}). (b) Polymer T_{cp} as a function of alanine content (200 µM, PBS) demonstrates the rise in critical temperature with alanine content and the slight depression in T_{cp} caused by the inclusion of oligoalanines in the POP sequences. (c) Optical density plots (200 µM, PBS) demonstrate the differences in T_{cp}-heating and T_{cp}-cooling caused by changing the mole fraction of helicity within POPs. Increasing helicity reduces T_{cp} and increases the range of the metastable hysteretic state. (d) Partial phase diagram for a mixture of ELP(V_1A_4) + POP(V)-25% illustrates the different discrete states available at different temperatures. If heating from 4 °C, POP will transition first followed by ELP, and cooling will result in dissolution of ELP first. 'Either' indicates that the protein is soluble upon heating from a lower temperature, but remains aggregated upon cooling from a higher temperature. (e) Partial phase diagram for a mixture of ELP(V) + POP(V_1A_4)-12.5% illustrates the opposing system where heating from 4 °C will cause the ELP to coacervate first, followed by the POP. Because of the metastable hysteretic range, however, ELP will also dissolve first upon cooling. Source data are provided as a Source Data file

Protein	Lane
ELP(V)	1
ELP(V_4A_1)	2
ELP(V_1A_1)	3
POP(V)-12.5%	4
POP(V)-25%	5
POP(V_4A_1)-25%	6
POP(V_1A_1)-25%	7

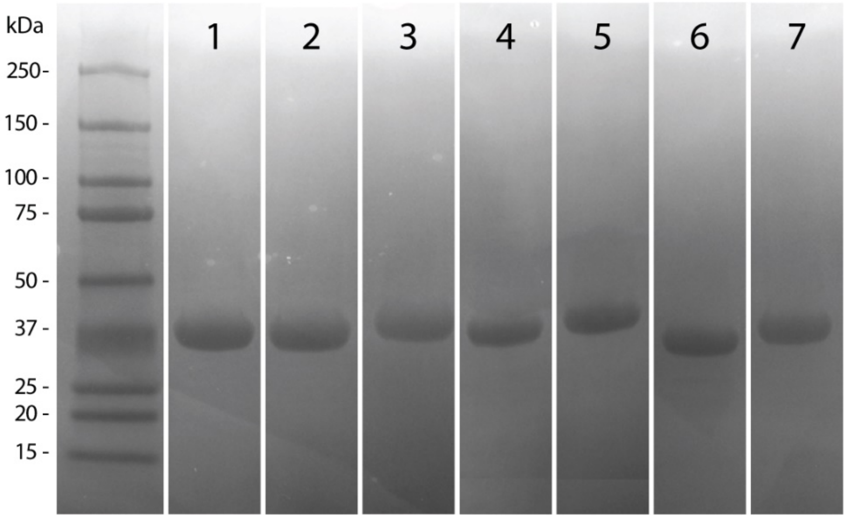

Supplementary Figure 2: Purity of protein polymers. All IDPs used were purified to >95% using ITC (Methods) as demonstrated by SDS-PAGE. Note that slight variations in predicted and actual molecular weight in SDS-PAGE gels are common for disordered proteins. Source data are provided as a Source Data file

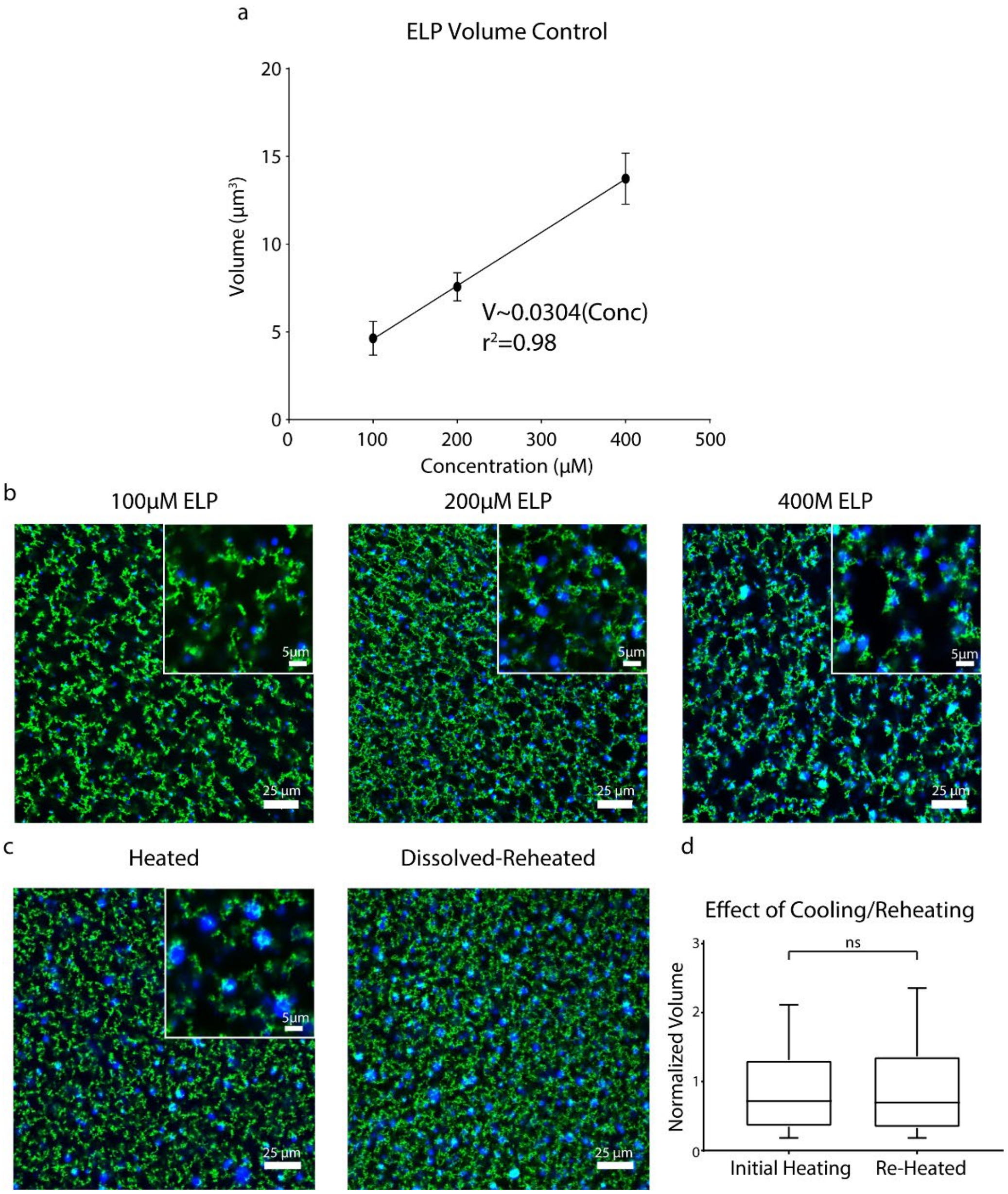

Supplementary Figure 3: Control of ELP globule size in bulk mixtures. (a) Confocal image analysis of ELP(V_4A_1) coacervate diameters when heated in PBS with POP(V)-25% (500μM) at different concentrations reveals that ELP coacervate volume is directly correlated with ELP concentration. Mixtures were prepared 3 separate times, and an n=150 of ELP "fruit" volumes were collected for each image (data represents mean ± sem) (b) Single plane images of ELP (blue) coacervates within a POP (green) networks show the change in size with concentration. (c,d) Because ELP aggregates after POP in this mixture, they can be cooled and reheated without change to the POP network. The average coacervate size after reheating is statistically identical (10-90% box and whiskers with median central line bounded by 25 and 75% quartiles, ns with $p>0.5$ as determined by two-tailed t-test, n=220 ELP 'fruits'). Source data are provided as a Source Data file

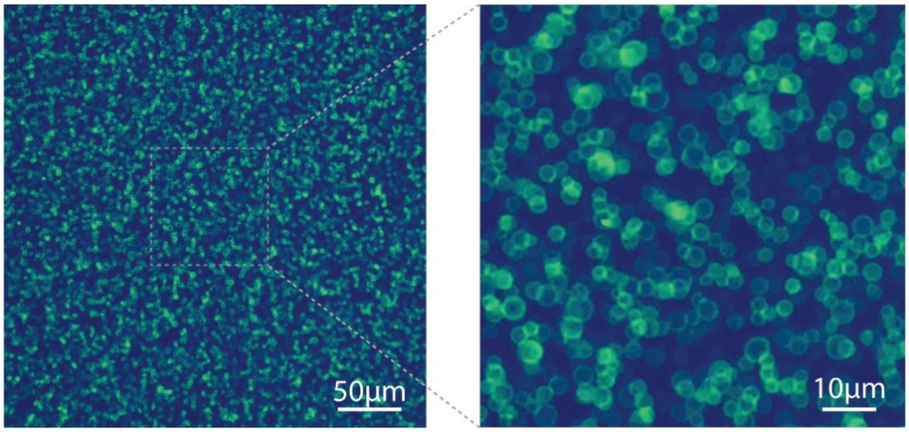

Supplementary Figure 4: Bulk images of hollow shell network. Fluorescent microscopy images of a mixture of ELP(V) (500 µM, blue) + POP(V_1A_4)-25% (100 µM, green) heated to > T_{cp} of both polymers and cooled to < T_{cp} of the ELP but within the metastable hysteretic range of the POP. The ELP has dissolved and diffused out of the cores, leaving an interconnected network of hollow POP shells.

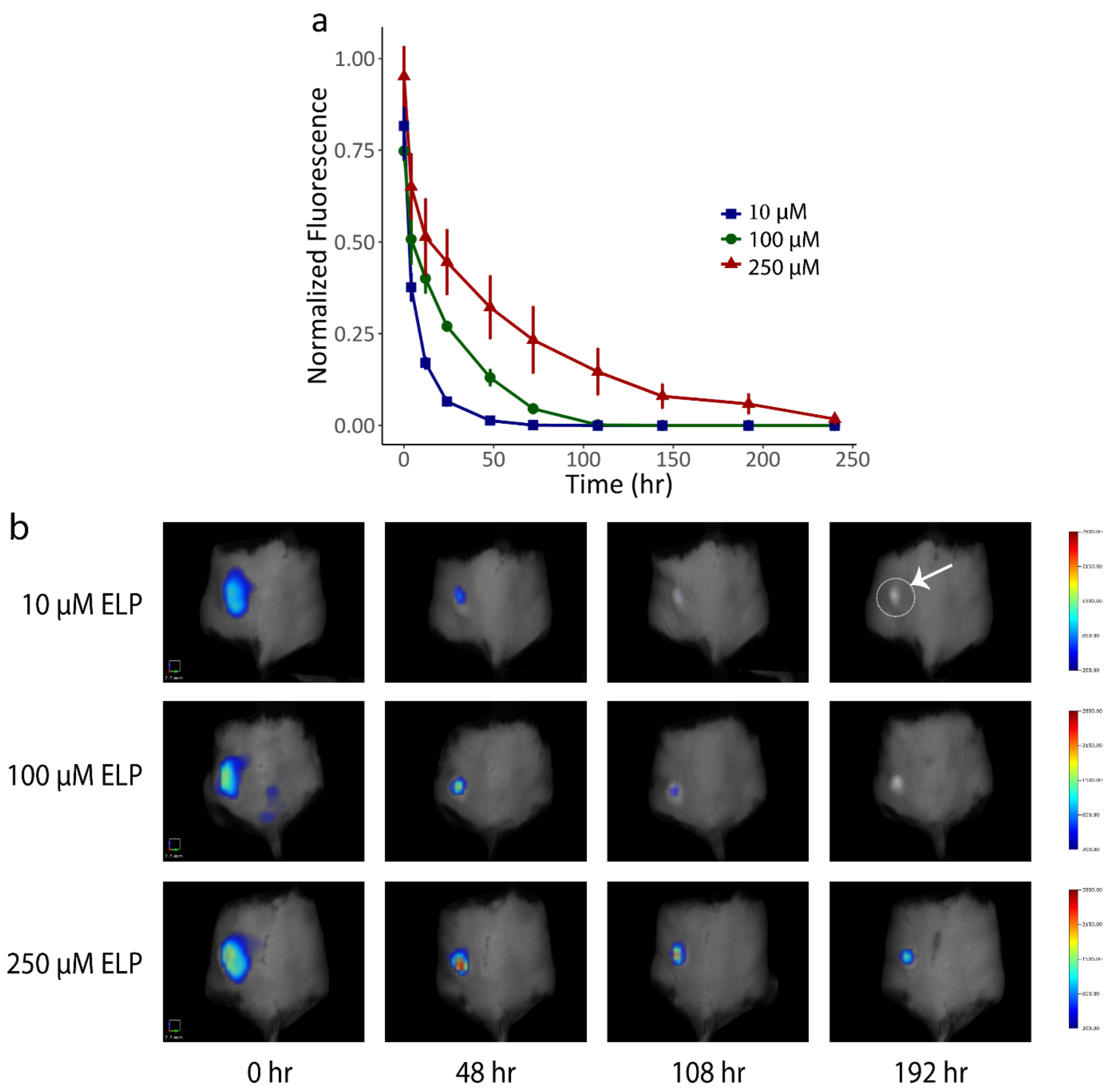

Supplementary Figure 5: Tunable sustained release of ELP from stable POP scaffolds. (a) Fluorescence molecular tomography (FMT) analysis of ELP(V_4A_1) co-injected along with POP(V)-25% subcutaneously in mice demonstrates the sustained-release of ELP from stable POP depots. Comparison between groups (n=5 mice, data represents mean ± sem) reveals that the duration of ELP retention is correlated with initial ELP concentration. (b) Representative FMT images show differences in ELP retention over time. Arrow notes POP depot which has remained at the site of injection despite elution of the ELP. Source data are provided as a Source Data file

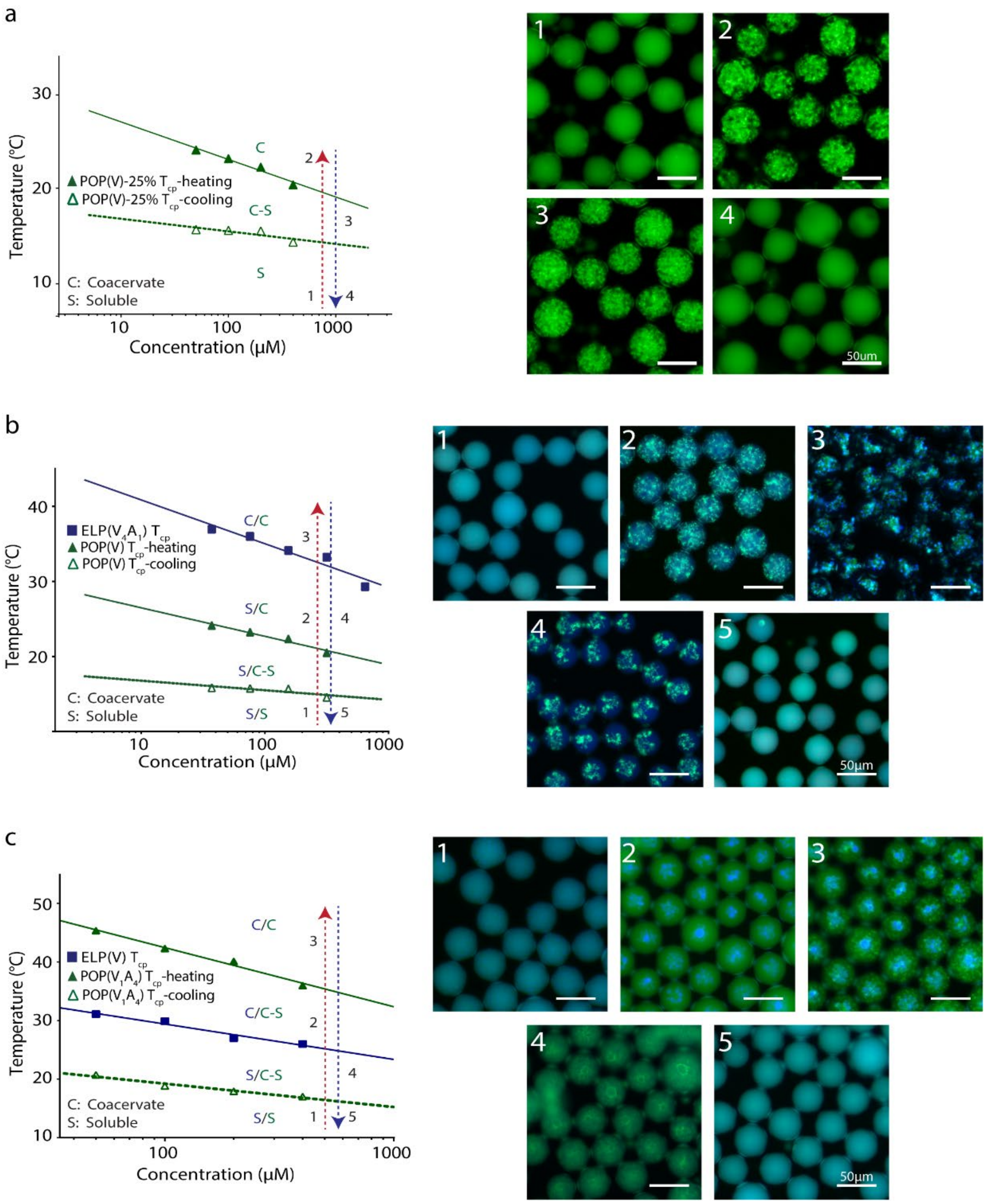

Supplementary Figure 6: Additional widefield images for microdroplet architectures. To demonstrate the uniformity of particle architectures across all particles during heat-cool cycles, reduced magnification images are provided to supplement the single particle images from Figure 2. The phase diagrams are duplicated here for reference. (a) Partial phase diagram for POP(V)-25% illustrating the different discrete states achievable during a heating and cooling cycle along with widefield fluorescent images of POP(V)-25% (500 µM) microdroplets during a heat-cool cycle. (b) Partial phase diagram for mixtures of POP(V)-25% (200 µM, green) + ELP(V_4A_1) (200 µM, blue) depicting the different states available during a heat-cool cycle of this system in which the POP aggregates first. Fluorescent images of each stage of the cycle demonstrate the formation of the fruits-on-a-vine architecture. (c) Partial phase diagram of ELP(V) (500µM) + POP(V_1A_4)-25% (100µM) depicting the states available during a heat-cool cycle and fluorescent images of each state of the cycle. Images here supplement the single particle images from Figure 2. Source data are provided as a Source Data file

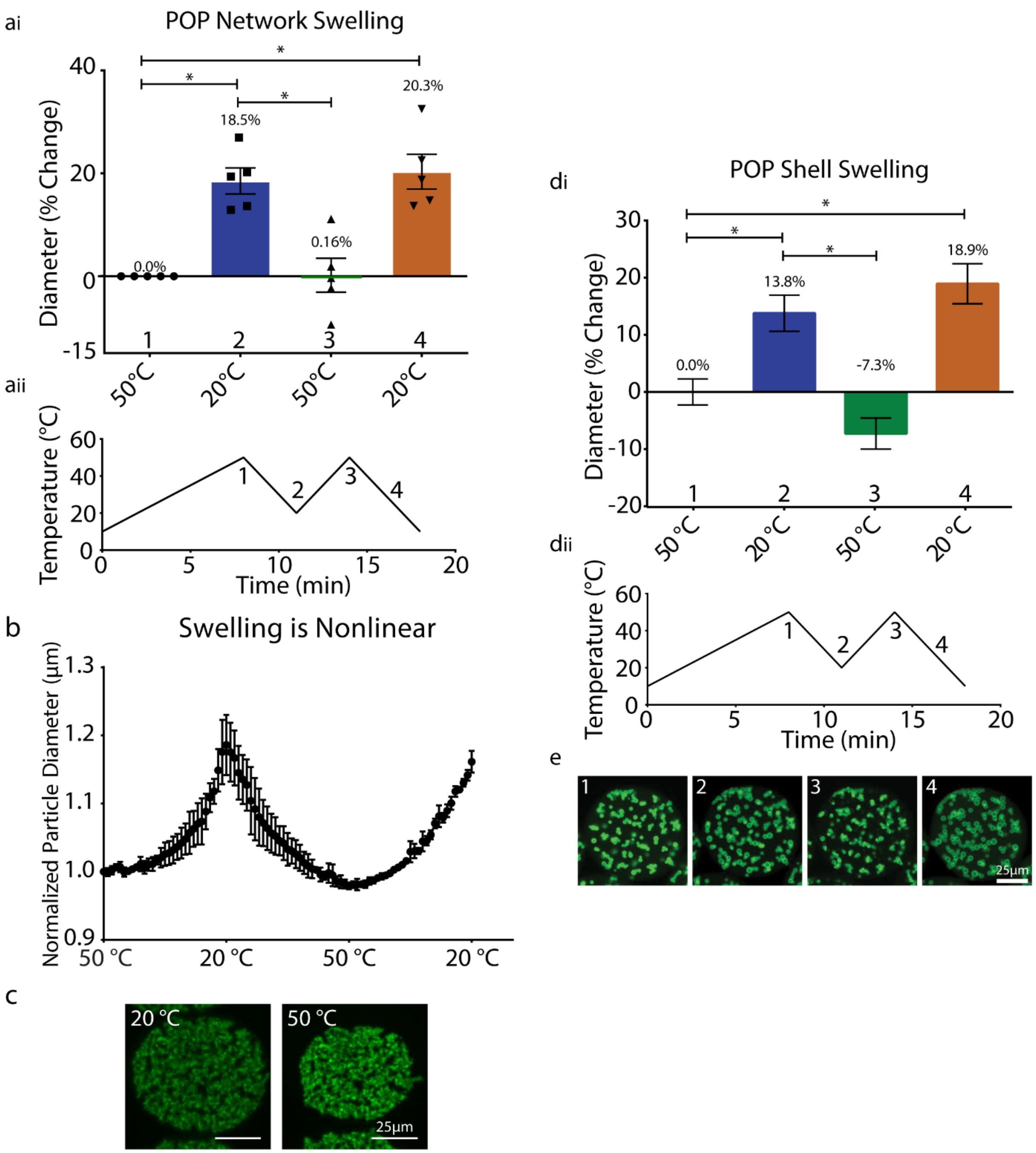

Supplementary Figure 7: POP particle shrinking and swelling. (a) POP(V)-25% (500 µM) was heated at 5°C/min to 50°C. Using the diameter at 50°C as a baseline, the swelling and shrinking was measured after cycling between 20°C and 50°C at 10°C/min (*$p<0.05$ as determined by repeated measures ANOVA with Tukey post hoc comparisons, n=5 particles, bar charts represent mean ± sem). (b) Videos of the particles were taken and size was analyzed over the course of the experiment (n=5 particles, data represents mean ± sem). The swelling is non-linear, approaching a minimum value at 50 °C. (c) Representative fluorescent microscopy images of the network show the difference in size at the minimum and maximum temperature. (d,e) Similar analysis was done with hollow POP shells after formation over ELP coacervates. Shells also swell at approximately ~20% (*$p<0.01$ as determined by repeated measures ANOVA with Tukey post hoc comparisons, n=130 shells, bar charts represent mean ± sem). Source data are provided as a Source Data file

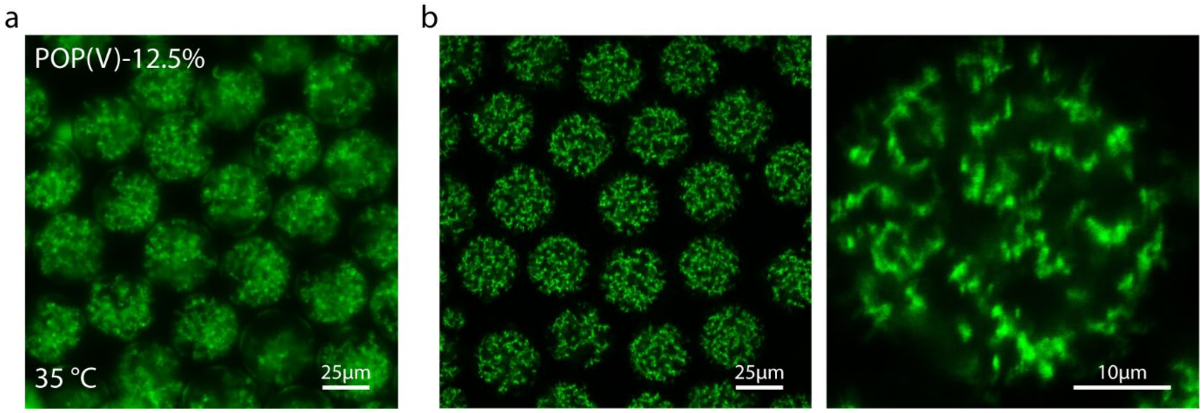

Supplementary Figure 8: 12.5% POP Particles. (a) Fluorescent and (b) confocal images of POP(V)-12.5% (500µM, PBS) indicate that they form identical architectures to that of 25% POPs.

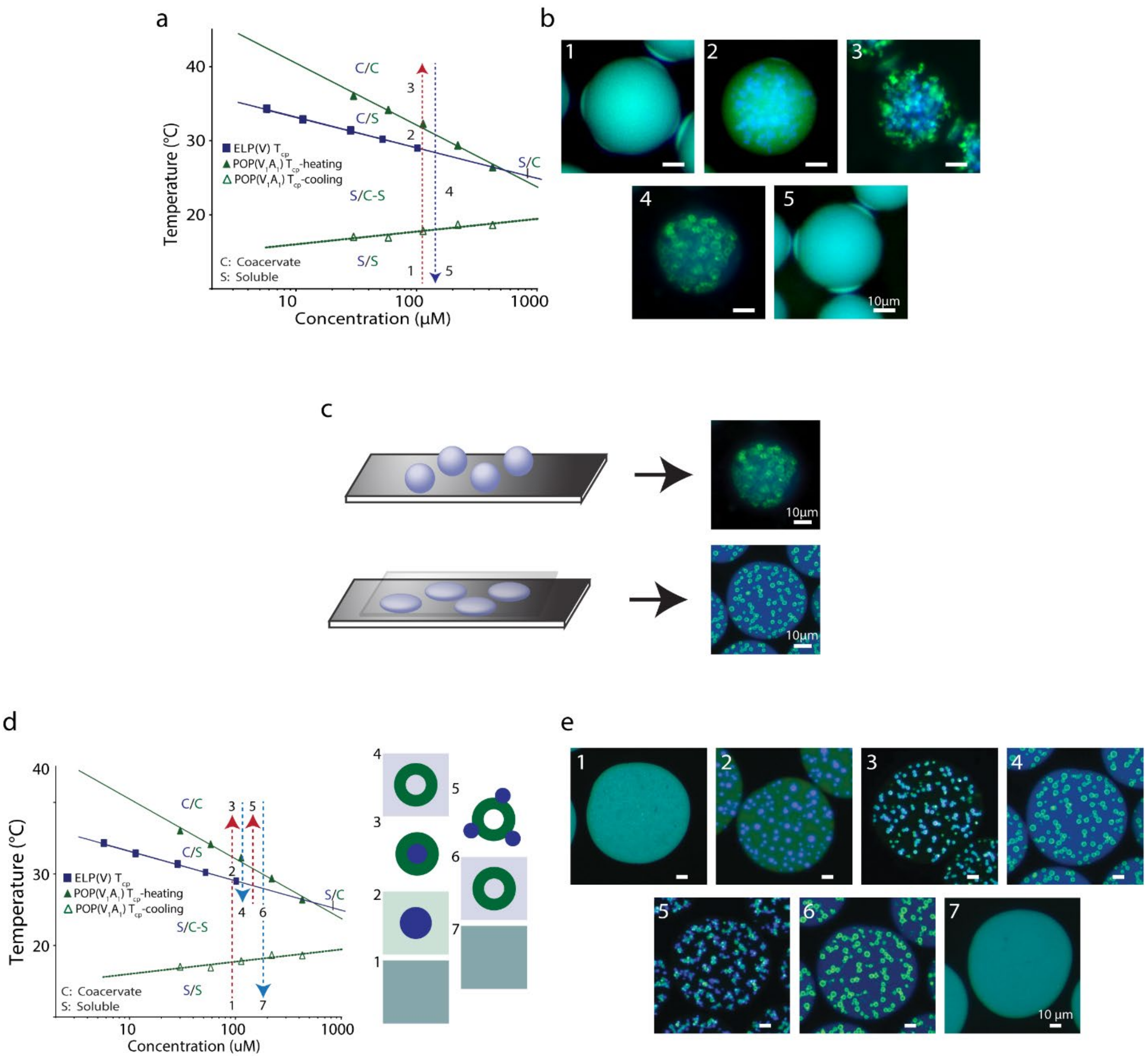

Supplementary Figure 9: Mixtures of ELP and a more hydrophobic POP. (b) Partial phase diagram of a mixture of ELP(V) (blue) + POP(V_1A_1)-25% (green) showing available states during a cycle of heating and cooling. (b) Fluorescent images of each stage for a mixture of ELP(V) (500 μM) + POP(V_1A_1)-25% (100 μM) show the formation of core-shell structures and hollow-shell structures similar to mixtures with POP(V_1A_4)-25% (Figure 2). (c) If compressed prior to aggregation with a coverslip, the core-shell and hollow shell structures are more visible, though they do not interconnect into a network. (d-e) The stages of aggregation and dissolution can be seen more clearly with no interconnection between POP shells. Notably, if the system is heated after dissolving out the ELP, ELP will aggregate and form globules which wet to the outside of the hollow shells (state 5). Source data are provided as a Source Data file

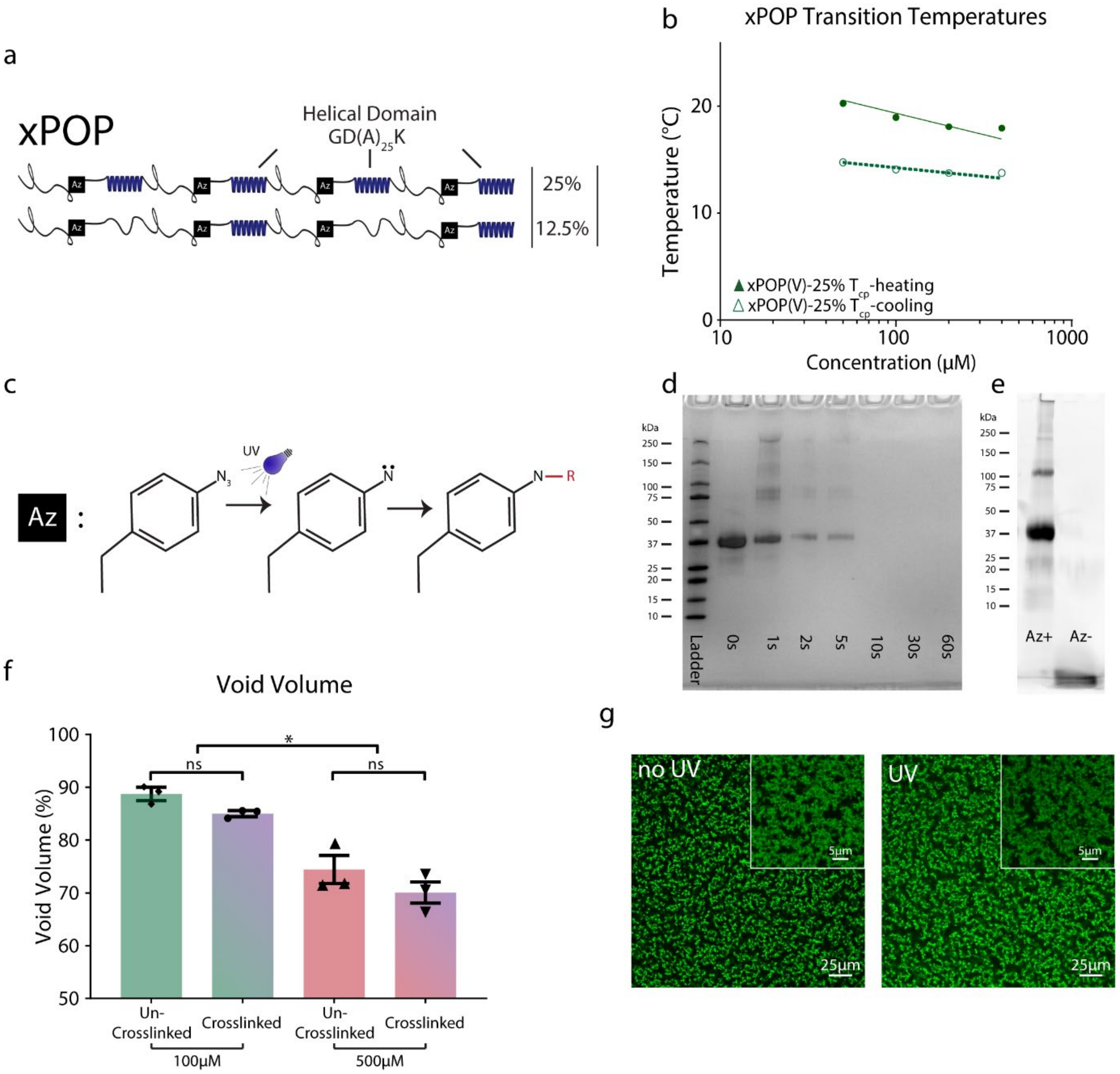

Supplementary Figure 10: Unnatural amino acids (UAA) for UV crosslinking. (a) xPOPs were created by encoding para-azidophenylalanine (*p*AzF) at 1 residue/100 amino acids. (b) Partial phase diagram for xPOPs shows that they retain both phase behavior and thermal hysteresis with a slightly downshifted T_{cp} due to the increased hydrophobicity of the aromatic UAA. (c) Depiction of the *p*AzF chemical changes during exposure of UV light. (d) SDS-PAGE analysis of xPOP(V)-25% crosslinking time. Polymer heated and then exposed to UV light for various times was subsequently cooled, centrifuged, and the resultant supernatant analyzed on an SDS-PAGE gel. Only soluble polymers not bound in a network remain in the supernatant and the eventual loss of bands (after 10s of UV exposure) indicates complete loss of un-networked polymer. (e) Fluorescent SDS-PAGE gel of xPOP(V)-25% labeled with DBCO-Cy5. *p*AzF residues react with DBCO-Cy5, while the negative control, which was expressed without the addition of *p*AzF to the media, is not fluorescently labeled via click chemistry. (f) Void volume (determined through analysis of reconstructed confocal images (n=3 unique networks, *p<0.05 as determined by one-way ANOVA with Tukey post hoc comparisons, bar charts represent mean ± sem) of xPOP(V)-25% remains un unchanged after UV crosslinking. (g) Confocal images (single plane with 5 µm stack in subset image) of uncrosslinked and UV crosslinked xPOP(V)-25% show no obvious differences in architecture. Source data are provided as a Source Data file

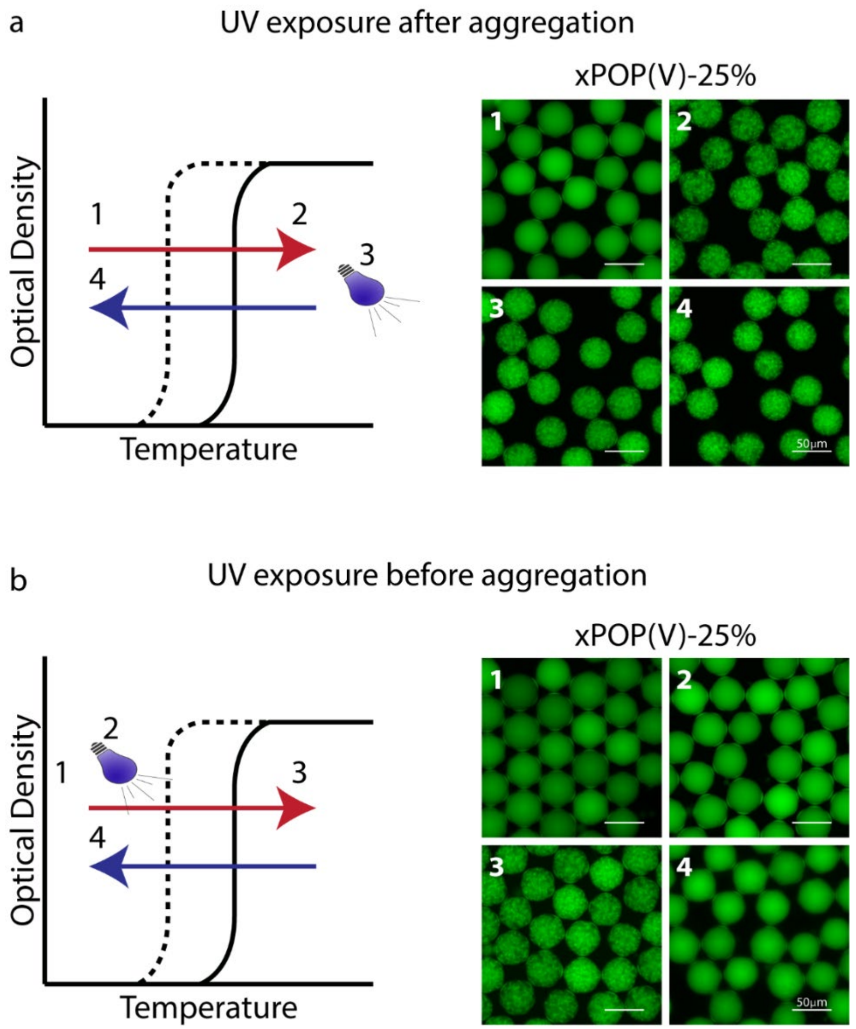

Supplementary Figure 11: UV exposure for xPOP microparticles. (a) xPOP(V)-25% (500 μM) particles crosslinked after aggregation and exposure to UV light do not solubilize upon cooling. (b) UV exposure prior to aggregation does not affect particle formation or reversibility.

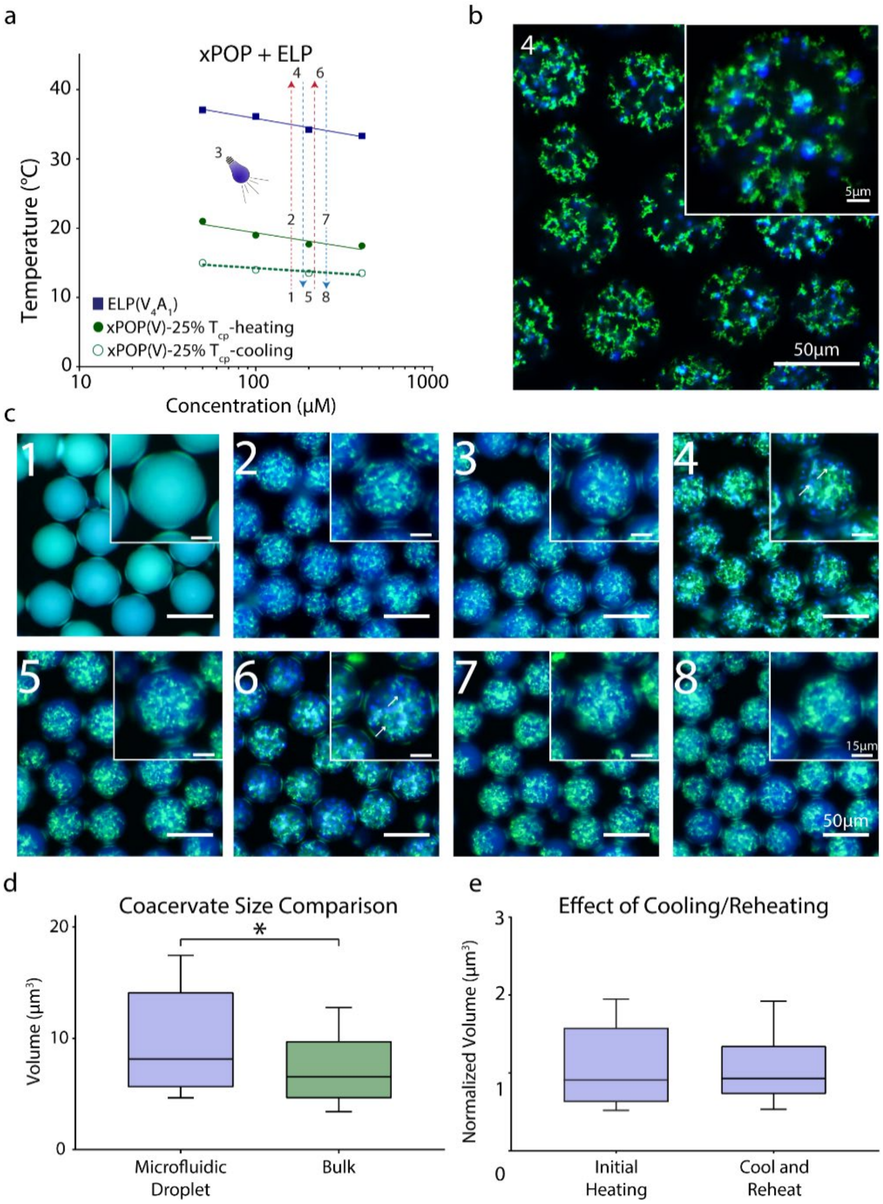

Supplementary Figure 12: xPOP mixture with a hydrophilic ELP. (a) Partial phase diagram of a mixture of xPOP(V)-25% + ELP(V_4A_1) showing the different states of coacervation achieved when progressing through a cycle of heating -> crosslinking -> cooling -> heating -> cooling. (b) A single plane confocal image of state 4 of the initial 'fruits-on-a-vine' architecture. (c) Fluorescent images of each state demonstrating the formation, dissolution, and re-formation of the ELP 'fruits' (blue) on a crosslinked xPOP particle scaffold (green). (d) Comparison of ELP 'fruits' formed in bulk and on microparticles formed from of a mixture of xPOP(V)-25% (200μM) + ELP(V_4A_1) (200μM), n=300 ELP 'fruits'. (e) Comparison of ELP 'fruits' formed on microparticles during initial heating and after dissolution and reheating; n=225 ELP 'fruits'. For d,e: 10-90% box and whiskers with median central line bounded by 25 and 75% quartiles, *p<0.01 as determined by two-tailed t-test. Source data are provided as a Source Data file

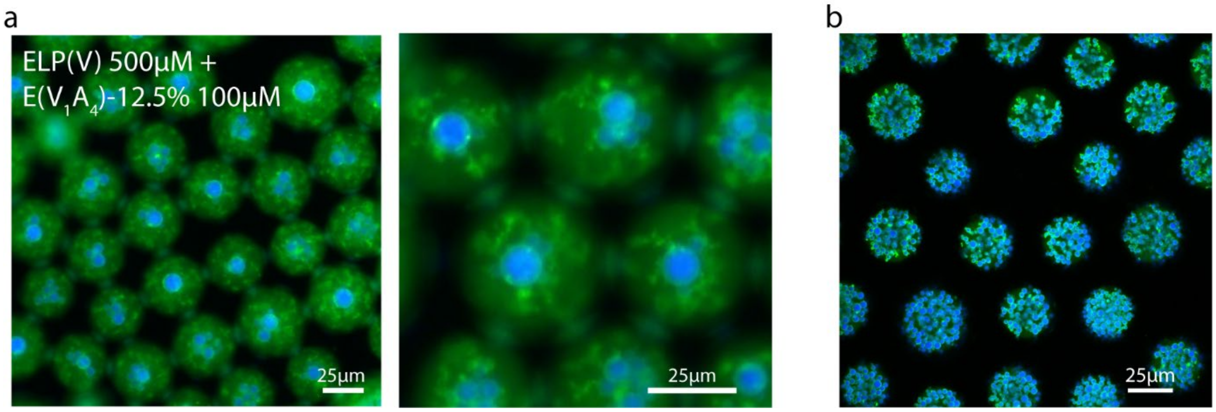

Supplementary Figure 13: 12.5% POP core-shell networks. (a) Fluorescent images of the core-shell structures created with ELP(V) (500 μM, blue) + POP(V_1A_4)-12.5% (100 μM, green). The resultant structures are similar to those formed with 25% POPs though the structures are slightly larger due to the more hydrophilic POP allowing greater time for ELP coacervation (heating rate 1°C/min for left/middle). (b) Confocal images of the same sample appear different as there no temperature ramp regulation.

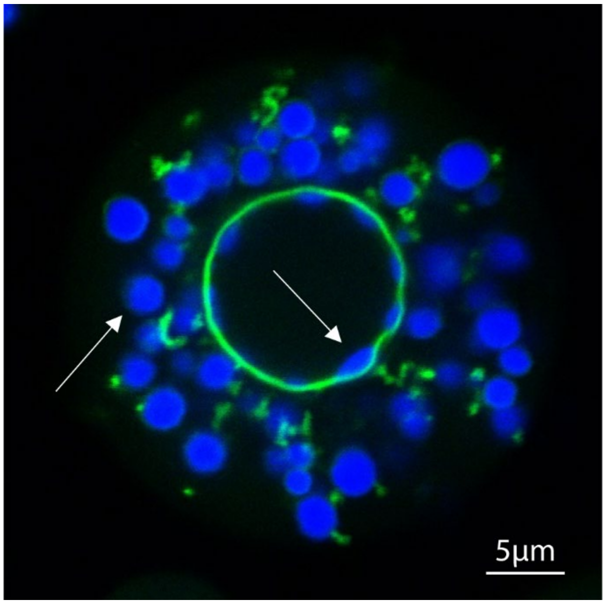

Supplementary Figure 14: ELP coacervation within POP shells. A single plane confocal image taken of a mixture of ELP(V) (1 mM, blue) + xPOP(V_1A_4)-12.5% (100 µM, green) after heating to form a core-shell structure, UV crosslinking to stabilize the shell, cooling to dissolve out the ELP, and finally reheating to re-coacervate the ELP. ELP does not refill the structure, though ELP left inside after diffusion will coacervate and collect on the inside of the xPOP shell.

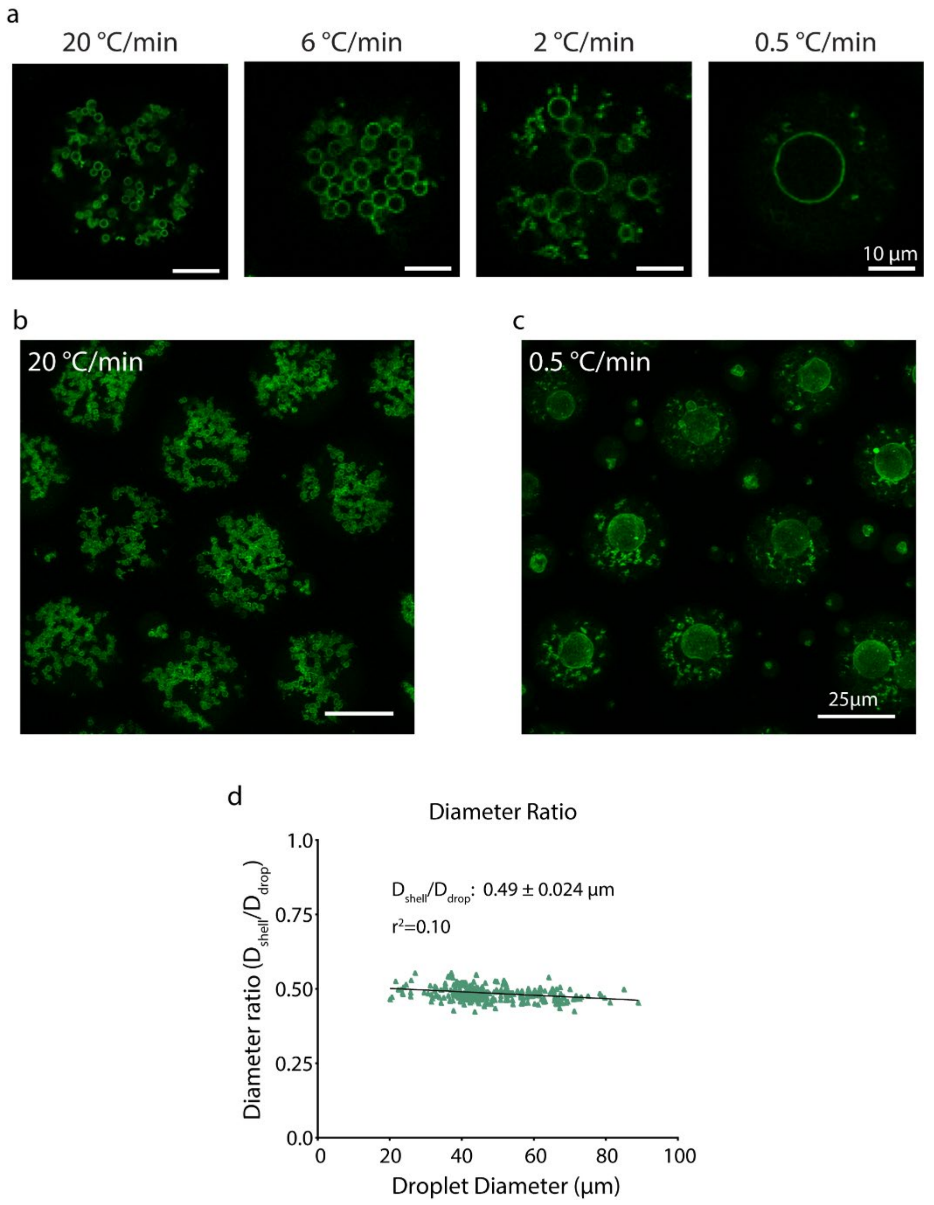

Supplementary Figure 15: Confocal reconstructions of hollow protein architectures. Mixtures of ELP(V) + xPOP(V_1A_4)-12.5% (100µM, green) were heated at different rates to produce different final architectures. (a) Single plane confocal images help elucidate the change in architecture as a function of heating rate depicted in Figure 3. 20 µm thick confocal stacks of resultant (b) core-shell networks and (c) single protein shells reveal their 3D architecture. Small protein aggregates outside of the structures arise due to minimal surface area for aggregation on ELP, but these are readily removed during extraction. (d) Linear regression analysis of a polydisperse mixture of ELP(V) (1mM) + xPOP(V_1A_4)-12.5% (100 µM) heated at 0.5 ^{o}C/min. The ratio of xPOP shell diameter to the diameter of the emulsion parallels remains constant regardless of emulsion size, providing a means by which to control xPOP shell size (n=280 droplets). Source data are provided as a Source Data file

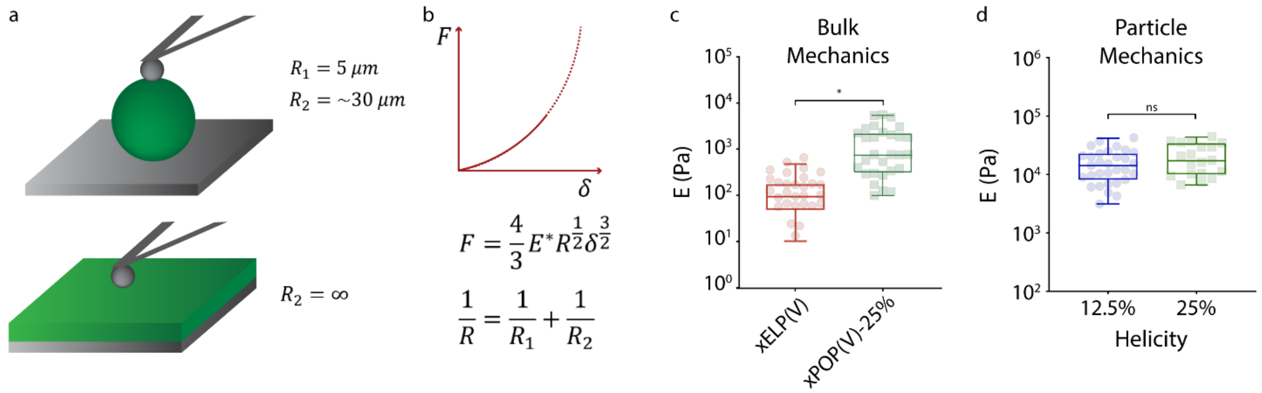

Supplementary Figure 16: Young's Modulus (E) of planar gels and POP microparticles. (a) For each microparticle and planar gel sample, a force curve was obtained along with an estimate of the particle radius from ImageJ analysis of AFM-recorded images, with R1=5 µm and R2=~30 µm for the bead format and R2=∞ for the planar gel format. (b) From each force *vs.* indentation ($F\ vs.\ \delta$) curve, E was obtained through curve fitting with the Hertz Model. For microparticles, each individual particle radius was used with its associated force curve during fitting. (c) UV crosslinked xELP(V) and xPOP(V) gels (1.5 mM) analyzed by AFM illustrate the increased mechanical integrity of POP networks compared to ELP hydrogels (10-90% box and whiskers with median central line bounded by 25 and 75% quartiles, *p<0.01 as determined by two-tailed t-test, n=30 depressions) (d) AFM analysis of extracted 500 µM POP microparticles revealed increased mechanical properties compared to bulk POP gels and no impact on the degree of helicity with E (10-90% box and whiskers with median central line bounded by 25 and 75% quartiles, ns with p>0.1 as determined by two-tailed t-test, n=30 12.5% particles and 20 25% particles). Source data are provided as a Source Data file.

Supplementary Table 1: Polymer Sequences

Polymer	Sequence
ELP(V)	$(GVGVP)_{80}$
ELP(V_4A_1)	$(G[V_4/A_1]GVP)_{80}$
ELP(V_1A_1)	$(G[V_1/A_1]GVP)_{80}$
ELP(V_1A_4)	$(G[V_1/A_4]GVP)_{80}$
POP(V)-25%	$((GVGVP)_{15}\text{-}GD(A)_{25}K)_4$
POP(V)-12.5%	$((GVGVP)_{35}\text{-}GD(A)_{25}K)_2$
POP(V_4A_1)-25%	$((G[V_4/A_1]GVP)_{15}\text{-}GD(A)_{25}K)_4$
POP(V_1A_1)-25%	$((G[V_1/A_1]GVP)_{15}\text{-}GD(A)_{25}K)_4$
POP(V_1A_4)-25%	$((G[V_1/A_4]GVP)_{15}\text{-}GD(A)_{25}K)_4$
POP(V_1A_4)-12.5%	$((G[V_1/A_4]GVP)_{35}\text{-}GD(A)_{25}K)_2$
xELP(V)	$((GVGVP)_{10}G\text{-}Az\text{-}GVP(GVGVP)_{10})_4$
xPOP(V)-25%	$((GVGVP)_5G\text{-}Az\text{-}GVP(GVGVP)_{10}GDA_{25}K)_4$
xPOP(V_1A_4)-25%	$(G[V_1\ /A_4]GVP)_5G\text{-}Az\text{-}GVP(G[V_1\ /A_4]GVP)_{10}GDA_{25}K)_4$
xPOP(V_1A_4)-12.5%	$((G[V_1\ /A_4]GVP)_{10}G\text{-}Az\text{-}GVP(G[V_1\ /A_4]GVP)_{15}G\text{-}Az\text{-}GVP(G[V_1\ /A_4]GVP)_{10}GDA_{25}K)_2$

*A Met leader and Gly-Try-Pro trailer are also include on all polymers

**Az = para-azidophenylalanine (*p* AzF)
