# Supplementary material for: Complex microparticle architectures from stimuli-responsive intrinsically disordered proteins
Source: Nat Commun. 2020 Mar 12;11:1342. doi: 10.1038/s41467-020-15128-9 (PMC7067844; doi:10.1038/s41467-020-15128-9)
Supplement: Supplementary file 3 — Reporting Summary [file 41467_2020_15128_MOESM3_ESM.pdf]

nature research

Corresponding author(s): Dr. Ashutosh Chilkoti
Last updated by author(s): Jan 29, 2020

Reporting Summary

Nature Research wishes to improve the reproducibility of the work that we publish. This form provides structure for consistency and transparency in reporting. For further information on Nature Research policies, see Authors & Referees and the Editorial Policy Checklist.

Statistics

For all statistical analyses, confirm that the following items are present in the figure legend, table legend, main text, or Methods section.

n/a	Confirmed	
☐	☒	The exact sample size (n) for each experimental group/condition, given as a discrete number and unit of measurement
☐	☒	A statement on whether measurements were taken from distinct samples or whether the same sample was measured repeatedly
☐	☒	The statistical test(s) used AND whether they are one- or two-sided<br>*Only common tests should be described solely by name; describe more complex techniques in the Methods section.*
☒	☐	A description of all covariates tested
☐	☒	A description of any assumptions or corrections, such as tests of normality and adjustment for multiple comparisons
☐	☒	A full description of the statistical parameters including central tendency (e.g. means) or other basic estimates (e.g. regression coefficient) AND variation (e.g. standard deviation) or associated estimates of uncertainty (e.g. confidence intervals)
☐	☒	For null hypothesis testing, the test statistic (e.g. F, t, r) with confidence intervals, effect sizes, degrees of freedom and P value noted<br>*Give P values as exact values whenever suitable.*
☒	☐	For Bayesian analysis, information on the choice of priors and Markov chain Monte Carlo settings
☒	☐	For hierarchical and complex designs, identification of the appropriate level for tests and full reporting of outcomes
☒	☐	Estimates of effect sizes (e.g. Cohen's d, Pearson's r), indicating how they were calculated

Our web collection on statistics for biologists contains articles on many of the points above.

Software and code

Policy information about availability of computer code

Data collection	Only commercial software was used for data collection, including Cary WinUV 2.x (Agilent), ZEN blue/black (Zeiss), Asylum Research Software v16, TrueQuant 2018 (PerkinElmer), and Scandium Desktop 2011 (ResAlta).
Data analysis	No custom code was used to analyze data. Analysis was primarily done in Microsoft Excel 2016 and Graphpad Prism 8. Basic algorithmic and publicly available plugins for ImageJ 2018 and MATLAB 9 were also used as detailed in the supplementary information.

For manuscripts utilizing custom algorithms or software that are central to the research but not yet described in published literature, software must be made available to editors/reviewers. We strongly encourage code deposition in a community repository (e.g. GitHub). See the Nature Research guidelines for submitting code & software for further information.

Data

Policy information about availability of data

All manuscripts must include a data availability statement. This statement should provide the following information, where applicable:

- Accession codes, unique identifiers, or web links for publicly available datasets
- A list of figures that have associated raw data
- A description of any restrictions on data availability

The authors declare that all data supporting the findings of this study are available within the manuscript and its supplementary files and are available from the authors on reasonable request. Source data underlying Figures 1b, 2b/c/f/i, 3a/b/d/e and Supplemental Figures 1b/c, 2, 3a/d, 5a, 7a/b/d, 10b/d/f, 12a/d/e, 15d, 16c/d are further provided as a Source Data file.

Field-specific reporting

Please select the one below that is the best fit for your research. If you are not sure, read the appropriate sections before making your selection.

☒ Life sciences ☐ Behavioural & social sciences ☐ Ecological, evolutionary & environmental sciences

For a reference copy of the document with all sections, see nature.com/documents/nr-reporting-summary-flat.pdf

Life sciences study design

All studies must disclose on these points even when the disclosure is negative.

Sample size	For animal experiments, the resource equation method was used to determine the minimum number of mice for each group. For in vitro microscopy imaging, n was determined by the number of particles/globules/spheres captured within at least 3 independent imaging windows.
Data exclusions	None.
Replication	All microscopy and optical density experiments were repeated successfully ≥ 3 times. AFM imaging was replicated twice, and in vivo experiments were not replicated. All proteins were expressed at least 3 times with no observable differences between independent batches.
Randomization	For animal experiments, mice were randomized prior to handling, and all mice were treated with identical measures prior to selection. The need for clear labeling and sample delineation precluded the randomization of samples used for in vitro analysis. Where possible, random microscope fields of views were used for data collection to prevent biased imaging choices.
Blinding	Blinding was not relevant to this work as no experimental treatments requiring this method were used.

Reporting for specific materials, systems and methods

We require information from authors about some types of materials, experimental systems and methods used in many studies. Here, indicate whether each material, system or method listed is relevant to your study. If you are not sure if a list item applies to your research, read the appropriate section before selecting a response.

Materials & experimental systems

n/a	Involved in the study	
☒	☐	Antibodies
☒	☐	Eukaryotic cell lines
☒	☐	Palaeontology
☐	☒	Animals and other organisms
☒	☐	Human research participants
☒	☐	Clinical data

Methods

n/a	Involved in the study	
☒	☐	ChIP-seq
☒	☐	Flow cytometry
☒	☐	MRI-based neuroimaging

Animals and other organisms

Policy information about studies involving animals; ARRIVE guidelines recommended for reporting animal research

Laboratory animals	C57BL/6J female mice (8 weeks old) were used for in vivo protein tracking
Wild animals	None.
Field-collected samples	None.
Ethics oversight	The Duke University Institutional Animal Care and Use Committee (IACUC) approved and provided oversight for the use of animals within the manuscript.

Note that full information on the approval of the study protocol must also be provided in the manuscript.
